# Before Diagnosing IVIG‐Refractory Kawasaki Disease: Distinguishing Persistent KD Inflammation From AGEP‐Related Drug Fever

**DOI:** 10.1002/ccr3.73005

**Published:** 2026-06-19

**Authors:** Muhammad Abdullah Awan, Hammad‐ud‐din Raza

**Affiliations:** ^1^ Wah Medical College, National University of Medical Sciences Rawalpindi Pakistan; ^2^ Wah Medical College Rawalpindi Pakistan

Dear Editor,

We read with great interest the case report by Li et al. titled “Kawasaki Disease Accompanied by Acute Generalized Exanthematous Pustulosis,” published in Clinical Case Reports [1]. The authors describe a 4‐year‐old boy who presented with classical Kawasaki disease (KD) features, including fever, conjunctival congestion, strawberry tongue, cracked lips, extremity edema, cervical lymphadenopathy, and a scarlatiniform rash, and who later developed a pustular eruption consistent with acute generalized exanthematous pustulosis (AGEP) [1]. The report is clinically valuable because it reminds clinicians that pustular lesions in a child with KD should not always be interpreted as a direct cutaneous manifestation of KD.

However, one diagnostic point deserves further emphasis: persistent fever after initial IVIG therapy should not automatically be equated with IVIG‐refractory KD when AGEP is evolving simultaneously. In the reported case, conjunctival congestion and extremity edema improved after the first IVIG dose, but fever persisted while the rash evolved into white pustules on the abdomen, groin, and extremities [1]. The patient was then diagnosed with both IVIG‐refractory KD and AGEP and received a second IVIG dose plus methylprednisolone [1]. This sequence is understandable, but it raises an important interpretive issue because AGEP itself can present with fever, leukocytosis, eosinophilia, and systemic inflammatory features [1].

The distinction matters clinically. IVIG resistance in KD usually implies ongoing vasculitic inflammation with concern for coronary artery injury, whereas AGEP‐related fever reflects a drug‐induced inflammatory skin reaction. In Li et al.'s case, echocardiography on Days 2 and 9 of hospitalization revealed no coronary artery dilatation, and repeat echocardiograms at 4 and 6 weeks remained normal [1]. These reassuring cardiac findings do not exclude KD, but they make it especially important to clarify why persistent fever was attributed to IVIG‐refractory KD rather than to AGEP‐related systemic inflammation or drug fever.

Several features in the case support the need for this separation. The pustular eruption appeared after beta‐lactam antibiotic exposure, the eosinophil count rose markedly to 2.47 × 10^9^/L, the immunoglobulin E level was elevated, IL‐5 was markedly increased, and the authors diagnosed AGEP using the EuroSCAR criteria [1]. These details strengthen the AGEP diagnosis, but the manuscript does not provide a fully itemized EuroSCAR score, skin biopsy confirmation, patch testing, or lymphocyte stimulation testing. The authors appropriately acknowledge that skin biopsy was not performed [1]. Given this limitation, future similar reports should state which clinical and laboratory criteria were used to separate AGEP‐driven fever from refractory KD inflammation.

We suggest that case reports of KD‐AGEP overlap should include a structured assessment before labeling the child as IVIG‐refractory. This should include the timing of fever relative to IVIG administration, drug exposure, and pustule onset; serial CRP, WBC, eosinophil, platelet, and liver enzyme trends; EuroSCAR scoring details; dermatology review; skin biopsy where clinically feasible; and serial echocardiographic findings. These data would help determine whether further immunomodulation is being given for true KD refractoriness, AGEP‐related inflammation, or both.

This point is not merely semantic. Over‐labeling IVIG resistance may lead to additional IVIG or escalation therapy when the dominant driver of persistent fever is a drug reaction. Conversely, under‐recognizing refractory KD may delay treatment in a child at risk of coronary complications. A structured framework would therefore protect both sides of the diagnostic dilemma: avoiding unnecessary escalation while preserving aggressive treatment when coronary‐risk inflammation remains likely.

We also agree with Li et al. that AGEP can recur with future exposure to the causative drug [1]. Therefore, documentation of the suspected culprit medication is not only dermatologically relevant but also essential for future pediatric safety. In the present case, cefixime and amoxicillin‐clavulanate exposure occurred before the pustular eruption, and the authors suspected amoxicillin‐clavulanate based on drug risk and rash timing [1]. Future reports would be strengthened by clearer documentation of culprit‐drug probability assessment and post‐recovery allergy counseling for parents.

Overall, Li et al. present an important case showing that KD and AGEP can coexist and that pustular rash should not be automatically assigned to KD [1]. The broader lesson is that persistent fever in such overlap cases must be interpreted through both a cardiology and dermatology lens. Future reports should distinguish IVIG‐refractory KD from AGEP‐related drug fever using predefined clinical, laboratory, dermatologic, and echocardiographic markers.

## 1 Conclusion

This case effectively highlights the rare coexistence of KD and AGEP. However, in KD‐AGEP overlap, persistent fever after first‐dose IVIG should not automatically be interpreted as IVIG‐refractory KD. Future case reports should define whether persistent fever is driven by ongoing KD vasculitis, AGEP‐related systemic inflammation, or drug fever, supported by EuroSCAR details, dermatologic confirmation where feasible, inflammatory‐marker trends, and serial echocardiography.

## Author Contributions

**Muhammad Abdullah Awan:** conceptualization, data curation, formal analysis, project administration, writing – original draft, writing – review and editing, supervision. **Hammad‐ud‐din Raza:** data curation, formal analysis, writing – original draft, writing – review and editing.

## Funding

The authors have nothing to report.

## Ethics Statement

The authors have nothing to report.

## Consent

The authors have nothing to report.

## Conflicts of Interest

The authors declare no conflicts of interest.

## Data Availability

Data sharing not applicable to this article as no datasets were generated or analysed during the current study.
